# *Turnip mosaic virus* manipulates *DRM2* expression to regulate host CHH and CHG methylation for robust infection

**DOI:** 10.1007/s44154-022-00052-3

**Published:** 2022-08-04

**Authors:** Xiaoyun Wu, Mengzhu Chai, Jiahui Liu, Xue Jiang, Yingshuai Yang, Yushuang Guo, Yong Li, Xiaofei Cheng

**Affiliations:** 1 Key Laboratory of Germplasm Enhancement, Physiology and Ecology of Food Crops in Cold Region of Chinese Education Ministry, College of Agriculture, Northeast Agricultural University, Harbin, 150030 Heilongjiang China; 2 Key Laboratory of Molecular Genetics, Guizhou Academy of Tobacco Science, Guiyang, 550081 China; 3 College of Life Science, Northeast Agricultural University, Harbin, 150030 Heilongjiang China

**Keywords:** Counter defense, *DRM2*, Hypermethylation, RNA-directed DNA methylation, *Turnip mosaic virus*

## Abstract

DNA methylation is an important epigenetic marker for the suppression of transposable elements (TEs) and the regulation of plant immunity. However, little is known how RNA viruses counter defense such antiviral machinery. In this study, the change of DNA methylation in *turnip mosaic virus* (TuMV)-infected cells was analyzed by whole genome bisulfite sequencing. Results showed that the total number of methylated sites of CHH and CHG increased in TuMV-infected cells, the majority of differentially methylated regions (DMRs) in the CHH and CHG contexts were associated with hypermethylation. Gene expression analysis showed that the expression of two methylases (*DRM2* and *CMT3*) and three demethylases (*ROS3*, *DML2*, *DML3*) was significantly increased and decreased in TuMV-infected cells, respectively. Pathogenicity tests showed that the enhanced resistance to TuMV of the loss-of-function mutant of *DRM2* is associated with unregulated expression of several defense-related genes. Finally, we found TuMV-encoded NIb, the viral RNA-dependent RNA polymerase, was able to induce the expression of *DRM2*. In conclusion, this study discovered that TuMV can modulate host DNA methylation by regulating the expression of *DRM2* to promote virus infection.

**Supplementary Information:**

The online version contains supplementary material available at 10.1007/s44154-022-00052-3.

## Introduction

Methylation at the C-5 position of cytosine (C) is an important and conserved DNA modification in eukaryotes that is associated with the suppression of transposable elements (TEs) and control of gene expression. In plants, methylation occurs not only in the CG dinucleotide, but also at the in CHH and CHG trinucleotides, where H represents A, T, or C. In Arabidopsis, the de novo DNA methylation of CG, CHH, and CHG is catalyzed by DOMAIN REARRANGED METHYLTRANSFERASE 2 (DRM2) via the RNA-directed DNA methylation (RdDM) pathway (Zhang et al. 2018). Symmetric CG and CHG methylation is maintained by DNA METHYLTRANSFERASE 1 (MET1) and CHROMOMETHYLASE 3 (CMT3), respectively; while asymmetric CHH methylation is maintained by CMT2 or DRM2 depending on the chromatin context (Zhang et al. 2018). DNA demethylation is catalyzed by four DNA glycosylases/demethylase, namely, REPRESSOR OF SILENCING 1 (ROS1), DEMETER (DME), DEMETER-LIKE 2 (DML2), and DML3. ROS1, DML2 and DML3 are expressed in all vegetative tissues, while DME is expressed in companion cells of the female and male gametes.

Levels of DNA methylation vary among different cell types and different developmental stage, and also change when plants are subjected to biological and abiotic stresses (Viggiano and de Pinto 2017). It has already been known that DNA methylation is an important regulator in plant resistance against extracellular pathogens: methylation-defective plants show enhanced resistance to bacteria and biotrophic oomycetes, but are more susceptible to necrotrophic fungi, while demethylation-defective mutants display enhanced resistance to necrotrophic fungi, but increased susceptibility to biotrophic oomycetes and bacteria (Deleris et al. 2016; Yu et al. 2013; Dowen et al. 2012; Sánchez et al. 2016; Le et al. 2014; Huang et al. 2022a). DNA methylation also responses to the infection of intracellular parasites like viruses: DNA methylation is an important defense mechanism against DNA viruses by direct attenuating viral gene transcription (Raja et al. 2008), while hypomethylation enhances resistance to the invasion of RNA viruses (Leone et al. 2020; Diezma-Navas et al. 2019; Corrêa et al. 2020). Given the important role of methylation in defenses against DNA viruses, it is not surprising that proteins of begomoviruses, e.g., C2, C3, V2, C4, and βC1, can directly interfere DNA methylation cycle with varied strategies (Gui et al. 2022; Mei et al. 2020; Wang et al. 2014; Zhou 2021; Chen et al. 2020). Moreover, DNA methylation may be affected by virus-derived siRNAs (Diezma-Navas et al. 2019; Annacondia and Martinez 2021; Yang et al. 2016, 2020; Huang et al. 2022b). However, whether proteins of RNA viruses could manipulate methylation-related genes to directly modulate DNA methylation is still elusive.

The genus *Potyvirus* within the family *Potyviridae* contains ~ 30% of the currently known plant RNA viruses including many destructive ones, such as *turnip mosaic virus* (TuMV), *soybean mosaic virus* (SMV), *plum pox virus* (PPV), *papaya ringspot virus* (PRSV), and *sugarcane mosaic virus* (SCMV) (Yang et al. 2021). The genome of potyviruses consists of a positive-sense single-stranded (+ ss) RNA of about 97,000-nucleotides-long, which encodes a total number of 11 proteins through two polypeptides (Revers and García 2015). Most potyviral proteins are multi-functional, which allow them to efficiently subvert varied host resistances (Yang et al. 2021). In this study, the defense and counter-defense in the context of DNA methylation was analyzed using TuMV-Arabidopsis as a model pathosystem. Our results showed that TuMV is able to regulate the expression of *DRM2* for modulating DNA methylation.

## Results

### Methylome in TuMV-infected cells

In order to investigate the change of DNA methylation in TuMV-infected cell, four-week-old *Arabidopsis thaliana* ecotype Col-0 (hereafter Arabidopsis) seedlings were mechanically inoculated with TuMV-GFP, a TuMV infectious clone expressing a free green fluorescent protein (GFP) between the P1 and HC-Pro cistrons for directly visualizing virus infection (Lellis et al. 2002). As a control, Arabidopsis seedlings of the same age were treated with inoculation buffer only. At 14 days post-inoculation (dpi), TuMV-infected areas of systemic leaves as indicated by GFP fluorescence were collected for genomic DNA isolation. Three replications of mock and TuMV-infected samples were applied, respectively. Genomic DNAs were treated with sodium bisulfite to convert unmethylated cytosines into uracil and then sequenced. After filtering of low-sequencing quality and duplication reads, a total of 126,055,542 (149 ×) and 123,831,204 (146 ×) unique reads from mock and TuMV-infected leaf tissues were obtained, respectively. Reads were mapped to Arabidopsis genome (TAIR10) and used for methylation calling. A total number of 41,057,244 and 41,615,743 cytosine in the Arabidopsis genome were covered by the reads from mock-treated and TuMV-infected Arabidopsis leaf tissues, respectively. Approximately 14.8% and 14.1% of the covered cytosine of mock and TuMV-infected Arabidopsis leaf tissues were respectively methylated.

**Fig. 1:**
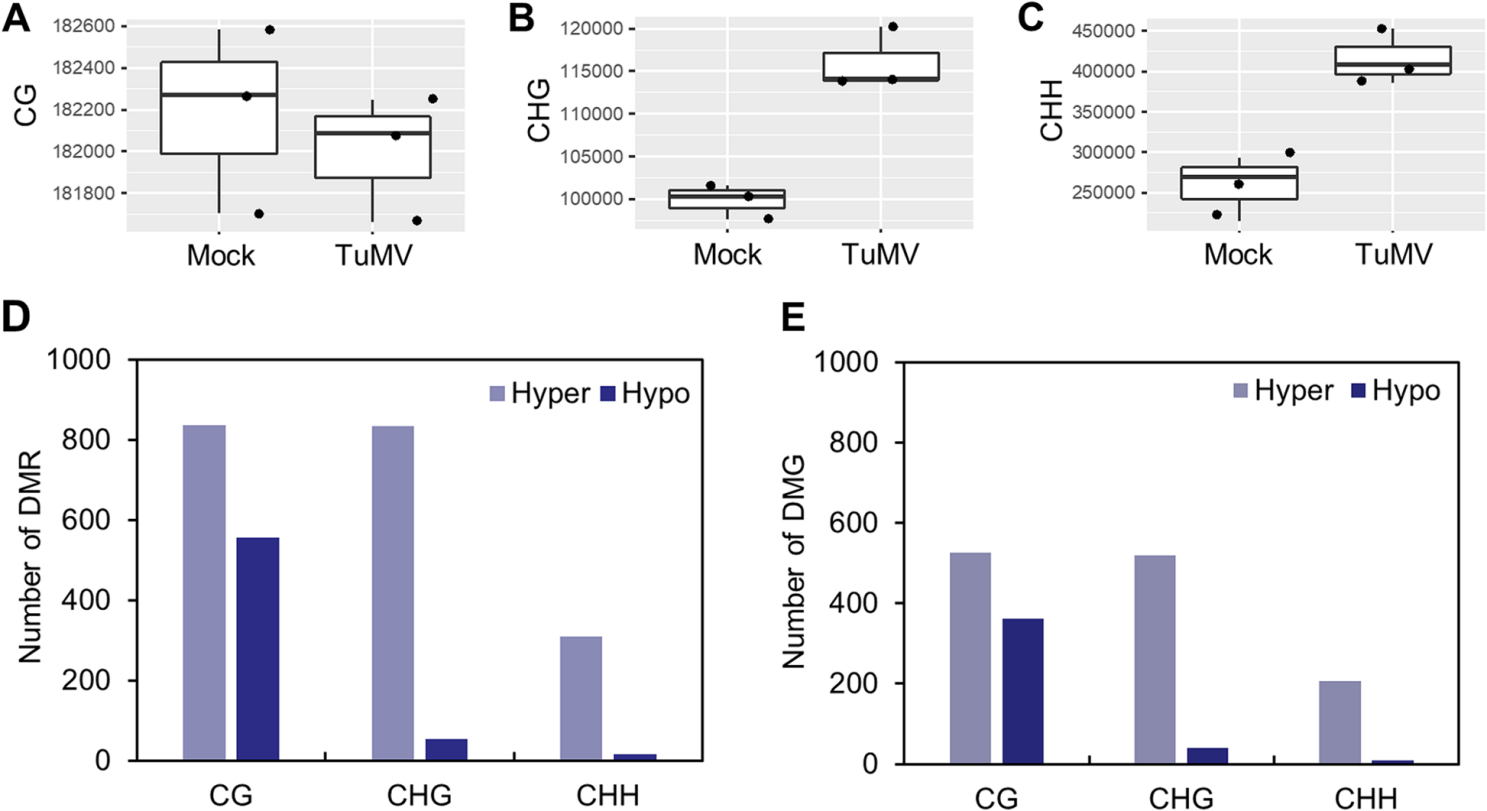
Whole-genome bisulfite sequencing (WGBS) of mock and TuMV-infected leaf tissues. **A**-**C** number of methylated sites of CG (**A**), CHG (**B**), and CHH (**C**), respectively. **D** Number of hyper- or hypomethylated differentially methylated regions (DMRs) in CpG, CHG, and CHH contexts. **E** Number of hyper- or hypomethylated DMRs-associated genes (DMGs) in CpG, CHG, and CHH contexts

We firstly analyzed methylation sites (methylated coverage ≥ 3, methylation rate ≥ 5%) in control and TuMV-infected leaf tissues. Results showed that TuMV infection caused the increment of CHH (*p* = 0.006) and CHG (*p* = 0.007) methylation but not the CG methylation (Fig. 1A-C). Differentially methylated regions (DMRs) in each methylation context were then identified. The infection of TuMV resulted in compatible number of hypermethylated and hypomethylated in the CG context (Fig. 1D; Table 1; Supplementary Files 1 and 2). However, DMRs of CHG and CHH were mostly belonged to the hypermethylation (Fig. 1D; Table 1; Supplementary Files 3, 4, 5 and 6). DMR-associated genes (DMGs) of the CG context were compatible, while DMGs of CHG and CHH were mostly associated with hypermethylation (Fig. 1E; Table 1). Kyoto Encyclopedia of Genes and Genomes (KEGG) pathway analysis showed that DMGs of CG were enriched in mRNA metabolism, chromatin organization, and negative regulation of gene expression. However, DMGs of CHH and CHG did not displayed a special enrichment in the KEGG pathway analysis. To check if hyper-methylation in TuMV-infected samples were caused by virus-derived small RNAs (vsiRNAs), the genome of TuMV-GFP was spliced into 24-nt fragments and then searched against Arabidopsis genome with a maximum of 4 nucleotide (nt) mismatch. Only one gene, *AUXIN RESPONSE FACTOR* 7 (*ARF7*) was identified as the potential candidate of vsiRNAs. Together, these results indicate that CHG and CHH mainly undergoes hypomethylation in TuMV-infected cells, while CG endures both hypermethylation and hypomethylation.

**Table 1:** Number of DMRs and DMGs

Context	DMRs total	DMRs hyper	DMRs hypo	DMGs total	DMGs hyper	DMGs hypo
CpG	1392	836	556	862	526	361
CHG	888	835	53	549	519	41
CHH	325	309	16	216	207	10

### TuMV infection changed the expression of methylation-related genes

**Fig. 2:**
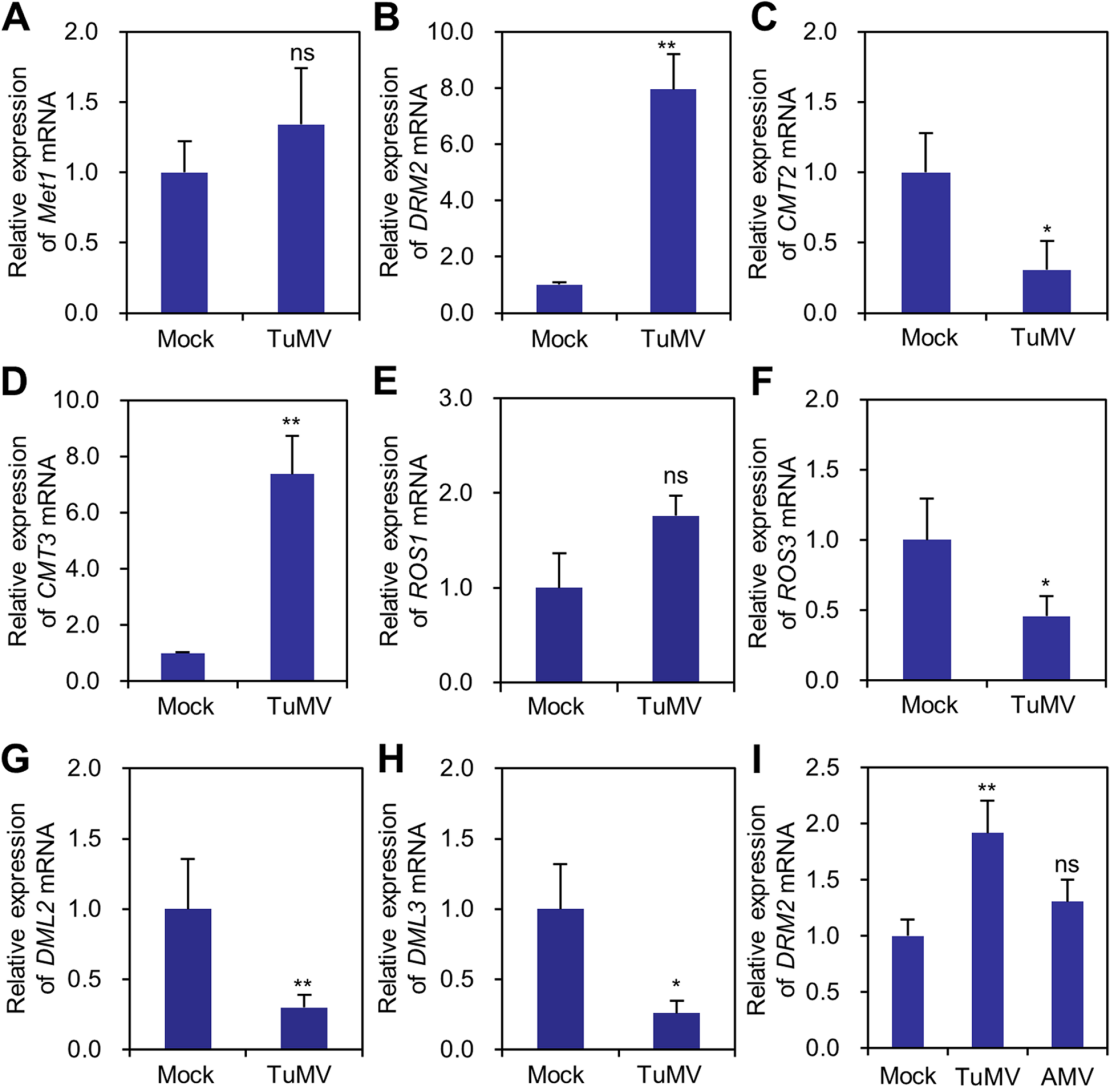
RT-qPCR analysis the expression of DNA methylation-related genes in mock and TuMV-infected leaf tissues. **A-H** The expression of *MET1* (**A**), *DRM2* (**B**), *CMT2* (**C**), *CMT3* (**D**), *ROS1* (**E**), *ROS3* (**F**), *DML2* (**G**), *DML3* (**H**) in mock and TuMV-infected leaf tissues, respectively. **I** RT-qPCR analysis the influence of TuMV and AMV infection to the expression of *DRM2*. *ACTIN II* was used as the internal control. Transcript level in mock plants was normalized to 1. Data are mean ± s.d. (*n* = 5)

We analyzed the expression of genes that are involved in DNA de novo methylation and/or maintenance, e.g., *DRM2*, *MET1*, *CMT2*, and *CMT*3, in mock or TuMV-infected leaf tissues by reverse transcription and quantitative PCR (RT-qPCR). We found that the expression of *DRM2* and *CMT3* in systemic TuMV-infected cells was significantly higher than that in mock plants, while the expression of *CMT2* and *MET1* was slightly reduced or unchanged in TuMV-infected plants at 14 dpi (Fig. 2A-D). We also analyzed the expression of demethylases including *ROS1*, *ROS3*, *DML2*, and *DML3* by RT-qPCR. The expression of *ROS3*, *DML2*, and *DML3* was significantly reduced in TuMV-infected leaf tissues, while the expression of *ROS1* was slightly increased in TuMV-infected leaf tissues (Fig. 2E-H). Thus, the elevated methylation content of CHH and CHG after the infection of TuMV may be associated with the upregulation of *DRM2* and *CMT3* and the downregulation of *ROS3*, *DML2* and *DML3*.

Since de novo methylation is exclusively catalyzed by *DRM2*, we focused our study on *DRM2*. To confirm the transcriptional change of DNA methylation-related genes is TuMV-specific, we inoculated four-week-old Arabidopsis seedlings by TuMV-GFP and *alfalfa mosaic virus* (AMV), an RNA virus of the genus *Alfamovirus* of the family *Bromoviridae*, and analyzed the expression of *DRM2* in the virus-infected leaf tissue at 14 dpi. We found that the expression level of *DRM2* in AMV-infected plants was comparable to mock plants at 14 dpi, while it was significantly upregulated in TuMV-infected plants at the same time point (Fig. 2B and I).

### Knock-out mutant of *DRM2* displayed increased resistance to TuMV

**Fig. 3:**
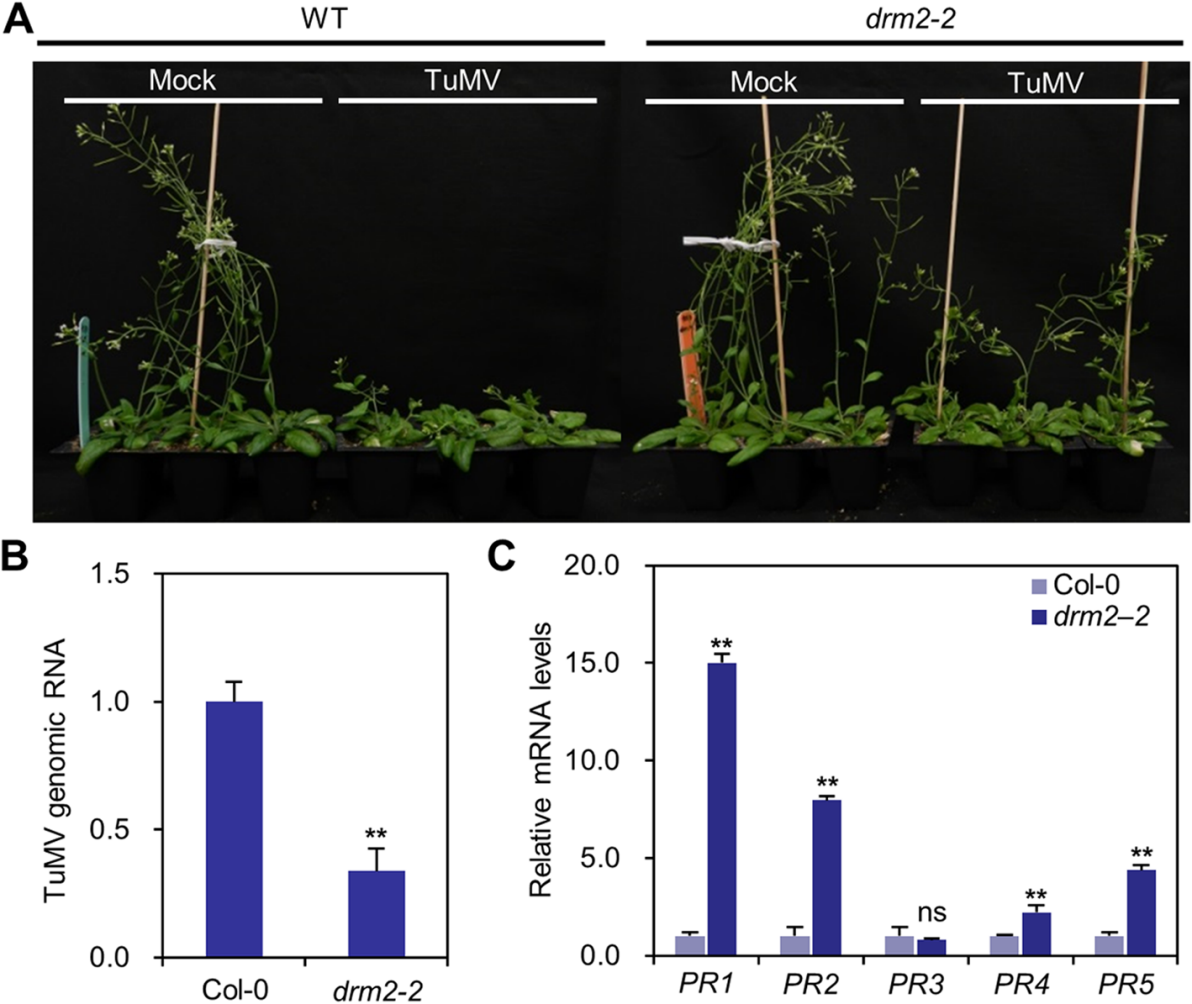
The *drm2–2* mutant displayed increased resistance to TuMV. **A** Phenotypes of TuMV-GFP on wild-type and *drm2–2* mutant at 20 dpi. **B** Relative levels of TuMV genomic RNA in wild-type and *drm2–2* mutant at 20 dpi. **C** Relative expression of *PR1*, *PR2*, *PR3*, *PR4*, and *PR5* in wild-type and *drm2–2* mutant at 20 dpi

To understand the biological function of *DRM2* in TuMV infection, we obtained a null mutant of *DRM2* (*drm2–2*; SALK_150863), which contain a T-DNA insertion in the predicted methyltransferase domain (Chan et al. 2006). Four-week-old seedlings of *drm2–2* or wild-type Arabidopsis were mechanically inoculated with TuMV-GFP. The plants were maintained in a growth chamber to monitor symptom development for a period of 20 days. At 20 dpi, wild-type Arabidopsis plants inoculated by TuMV-GFP showed typical TuMV infection symptoms, including mottling rosette leaves and stunted inflorescence stems, whereas viral symptoms on rosette leaves of *drm2–2* plants were much milder and the length of inflorescence stems were significantly increased (Fig. 3A). Furthermore, RT-qPCR showed that viral genomic RNAs in the *drm2–2* plants reduced by approximately 60% as compared with that in wild-type plants infected by TuMV at 20 dpi (Fig. 3B). Knock-out of *DRM2* causes DNA hypomethylation, which results in the upregulated of genes that controlled by methylation (Cao et al. 2003). Therefore, we analyzed the expression level of defense-related genes, e.g., *PATHOGENESIS-RELATED 1* (*PR1*), *PR2*, *PR3*, *PR4*, and *PR5* in TuMV-infected seedlings of *drm2–2* or wild-type Arabidopsis by RT-qPCR. We found that the expression levels of *PR1*, *PR2*, *PR4*, and *PR5* in *drm2–2* that infected by TuMV were significantly higher than that in wild-type Arabidopsis plants infected by TuMV (Fig. 3C). Together these results suggest that the increased resistance to TuMV in *drm2–2* is likely related to the elevated expression level of a subset, if not all, of defense-related genes.

### The expressions of partial defense-related genes were increased in *drm2–2*

**Fig. 4:**
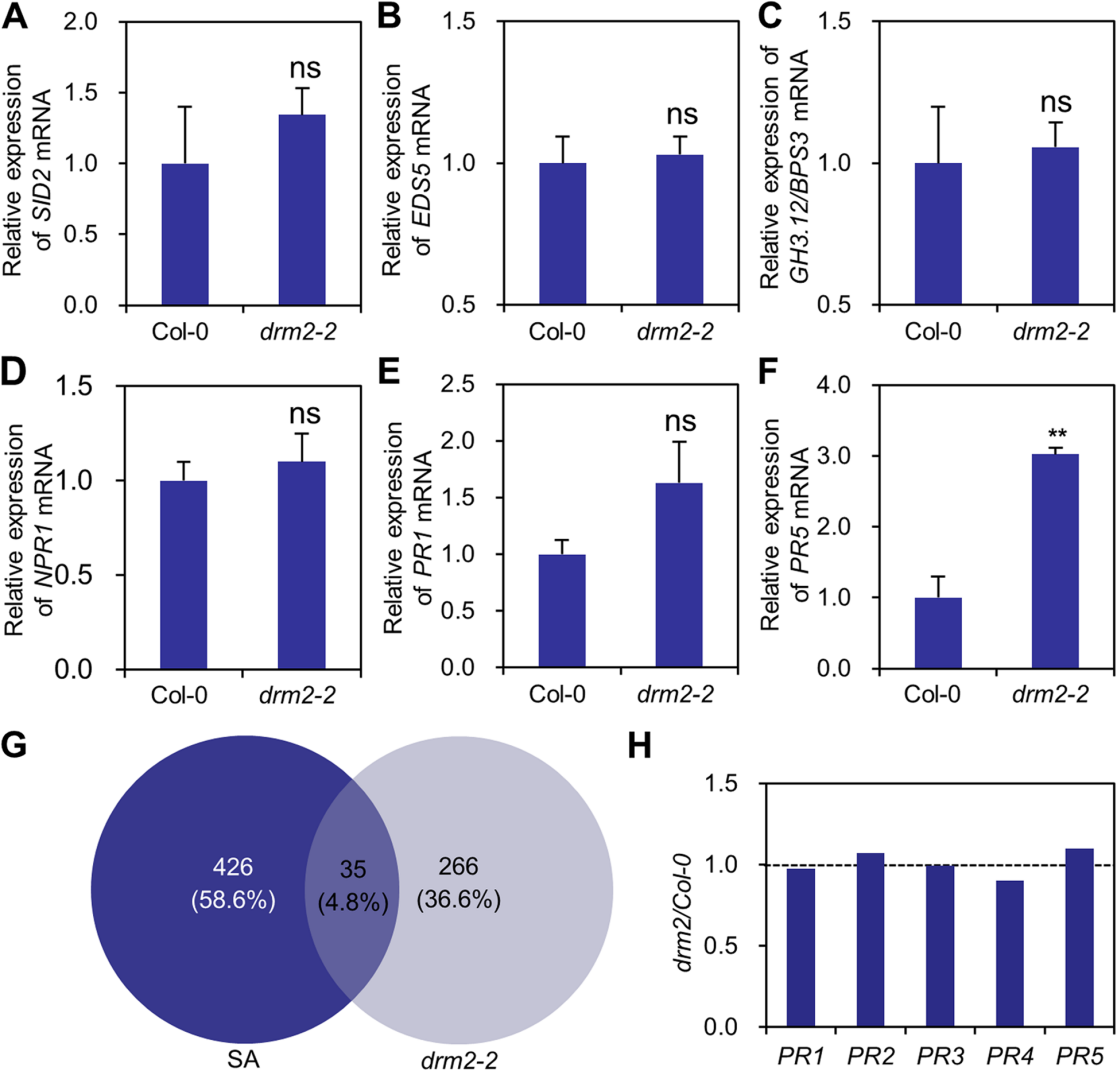
RT-qPCR and transcriptome analysis of the expression of salicylic acid-related genes. **A-F** The expression of *SID2* (**A**), *EDS5* (**B**), *GH3.12/PBS3* (**C**), *NPR1* (**D**), *PR1* (**E**), and *PR3* (**F**) in wild-type and *drm2–2* mutant, respectively. *ACTIN II* was used as the internal control. Transcript level in mock plants was normalized to 1. Data are mean ± s.d. (*n* = 5). **G** Venn diagram showing numbers of DEGs between mock- and SA-treated wild-type Arabidopsis (dark blue) and between wild-type Arabidopsis and *drm2* (light blue). **H** Abundant of *PR1-5* transcript in transcriptome of three-week-old seedlings of *drm2* and wild-type Arabidopsis

To further insight the mechanism of *DRM2*-mediated resistance, we compared the expression levels of genes that are involved in salicylic acid (SA) biogenesis, signaling, and that are regulated by SA, including *SA-DEFICIENT 2* (*SID2*; also known as *ISOCHORISMATE SYNTHASE 1*, *ICS1*), *ENHANCED DISEASE SUSCEPTIBILITY 5* (*EDS5*), *GRETCHEN HAGEN 3.12/AVRPPHB SUSCEPTIBLE 3* (*GH3.12*/*PBS3*; also known as *HopW1-1-interacting 3*, *WIN3*), *NONEXPRESSER OF PR GENES 1* (*NPR1*), *PR1*, and *PR3*. *EDS5*, *SID2*, and *GH3.12*/*PBS3* function in SA biosynthesis (Ding and Ding 2020), while *NPR1* is essential for the perception of SA and activation of the expression of downstream defense-related genes such as *PR1* and *PR5* (Chen et al. 2021; Mhamdi 2019). RT-qPCR results showed that the expression levels of *SID2*, *EDS5*, *GH3.12*/*PBS3*, *NPR1*, and *PR5* in *drm2–2* were similar to that in wild-type Arabidopsis plants (Fig. 4A-E). In contrast, the expression levels of *PR5* in *drm2–2* were significantly higher than in wild-type Arabidopsis plants (Fig. 4F). These results indicate that the enhanced resistance in *drm2–2* is not associated with elevated SA level.

We also downloaded published RNA-Seq data of the *drm2* mutant and SA induced Arabidopsis from NCBI SRA database (Table 2). Compared with mock plants, 461 differentially expressed genes (DEGs) and 301 DEGs were obtained from SA-treated plants and *drm2* mutants from 28,959 expressing genes of Arabidopsis, respectively (*p* < *0.05*). These results are consistent with previous studies (Stroud et al. 2014; Wang and Zhang 2021). Interestingly, 35 DEGs were shared by *drm2* and SA treatment (Fig. 4G; Supplementary File 1). KEGG pathway analysis showed that 35 DEGs were enriched in chloroplast stroma, chloroplast envelope, vacuole, lytic vacuole and apoplast (Supplementary File 7). The expression levels of five *PR* genes (*PR1*–*5*) were also compared between *drm2* mutant and wild-type Arabidopsis plants. Results showed that the expression of *PR2* and *PR5* was also increased in *drm2* as compared to that in wild-type plants (Fig. 4H). Together, these results indicate that knockout of *DRM2* causes the upregulation of a small proportion of SA-activated genes at steady conditions.

**Table 2:** Information of RNA-Seq data used for transcriptome analysis

Experiment	Sample	SRA accession	Description
*drm2*	Col-0	SRR1005385	RNA was extracted from leaf tissues of three-week-old seedlings of *drm2* or wild-type Arabidopsis Col-0
		SRR1005386	
	*drm2*	SRR1005387	
		SRR1005388	
SA	H_2_O treatment	ERR6800722	Leaves of Arabidopsis Col-0 were sprayed with water or 0.5 mM SA. At 24 h post spray, leaf tissues were harvested for RNA extraction
		ERR6800723	
	SA treatment	ERR6800712	
		ERR6800713	

### Viral RNA-dependent RNA polymerase induced *DRM2* expression

To explore viral protein(s) that can modulate the expression of DNA methylation-related genes, we transiently expressed the eleven proteins encoded by TuMV as N-terminal FLAG and 4 × Myc (FLAG-4 × Myc)-tagged recombinant proteins in *Nicotiana benthamiana* epidermal cells. At 2 dpi, transcript level of *N. benthamiana*-encoded *DRM2* (*NbDRM2*) was evaluated by RT-qPCR and the expression of each viral protein was validated by Western blot using anti-Myc antibodies. Results showed that the transcript level of *NbDRM2* was significantly increased in the leaves expressing NIb, the viral RNA-dependent RNA polymerase (RdRp). We further compared the transcript level of *DRM2* in wild-type Arabidopsis and transgenic plant expressing FLAG-4 × Myc-NIb. Results showed that the expression of *DRM2* was also increased in the two transgenic lines. Together, these results showed that NIb is able to module the expression of *DRM2*.

## Discussion

Previous studies showed that the infection of potyviruses, e.g., TuMV and SCMV caused comprehensive changes of DNA methylation in host plant (da Silva et al. 2020; Corrêa et al. 2020). In this study, we further analyzed the methylome change in virus-infected cells to insight the arms race between virus and host at the DNA methylation level. Our methylation analyses support the notion that DNA methylation levels are interfered by viral infection. Methylation- and demethylation-defective plants show enhanced resistance and susceptibility to RNA viruses, e.g., *tobacco rattle virus* (TRV), and *tobacco mosaic virus* Cg (TMV-Cg), respectively (Leone et al. 2020; Diezma-Navas et al. 2019). Thus, demethylation is a common response of plant to the infection of RNA viruses. Interestingly, our results showed an overall increment of CHH and CHG methylation level in TuMV-infected cells (Fig. 1A). The increased CHH and CHG methylation levels are associated with significant upregulation of *DRM2* and *CMT3*, and downregulation of *ROS3*, *DML2* and *DML3* (Fig. 2). In contrast, AMV infection did not induce *DRM2* expression (Fig. 2I), and the infection of TRV and *cucumber mosaic virus* (CMV) caused downregulation of DNA methylation-related genes, e.g., *MET1*, *DRM1*, and *CMT3* (Diezma-Navas et al. 2019; Wang et al. 2018). These results indicate that TuMV is able to modulate host DNA methylation possibly by regulating the expression of methylation-related genes.

KEGG pathway analysis showed that DMGs of CG were not particular enriched in plant-pathogen interaction, but in mRNA metabolism, chromatin organization, and negative regulation of gene expression, which is largely consistent with other studies using different phytosystems (Hewezi et al. 2017; Dowen et al. 2012; Sánchez et al. 2016). Loss-of-function of *DRM2* results in enhanced resistance to RNA viruses, biotrophic oomycetes, and bacteria (Deleris et al. 2016; Yu et al. 2013; Dowen et al. 2012; Sánchez et al. 2016; Le et al. 2014; Huang et al. 2022a; Leone et al. 2020; Diezma-Navas et al. 2019), suggesting that *DRM2* plays a critical role in plant immunity. RT-qPCR and transcriptome analyses revealed that the expression of some defense-related genes was upregulated in *drm2–2* even at the absence of virus infection as compared with that of wild-type Arabidopsis (Fig. 4). Moreover, the expression of *PR1*, *PR2, PR3*, and *PR5* was significantly higher than that in wild-type Arabidopsis (Fig. 3C). These results indicate that the enhanced resistance of *drm2* may be a directly consequence of changes in the expression of some SA-activated genes and/or a more rapid and intense the expression of *PR* genes during pathogen invasion. Given the importance of DNA methylation in plant immunity, it is promise to improve crop resistance by modulate the expression of *DRM2* (Tirnaz and Batley 2019).

**Fig. 5:**
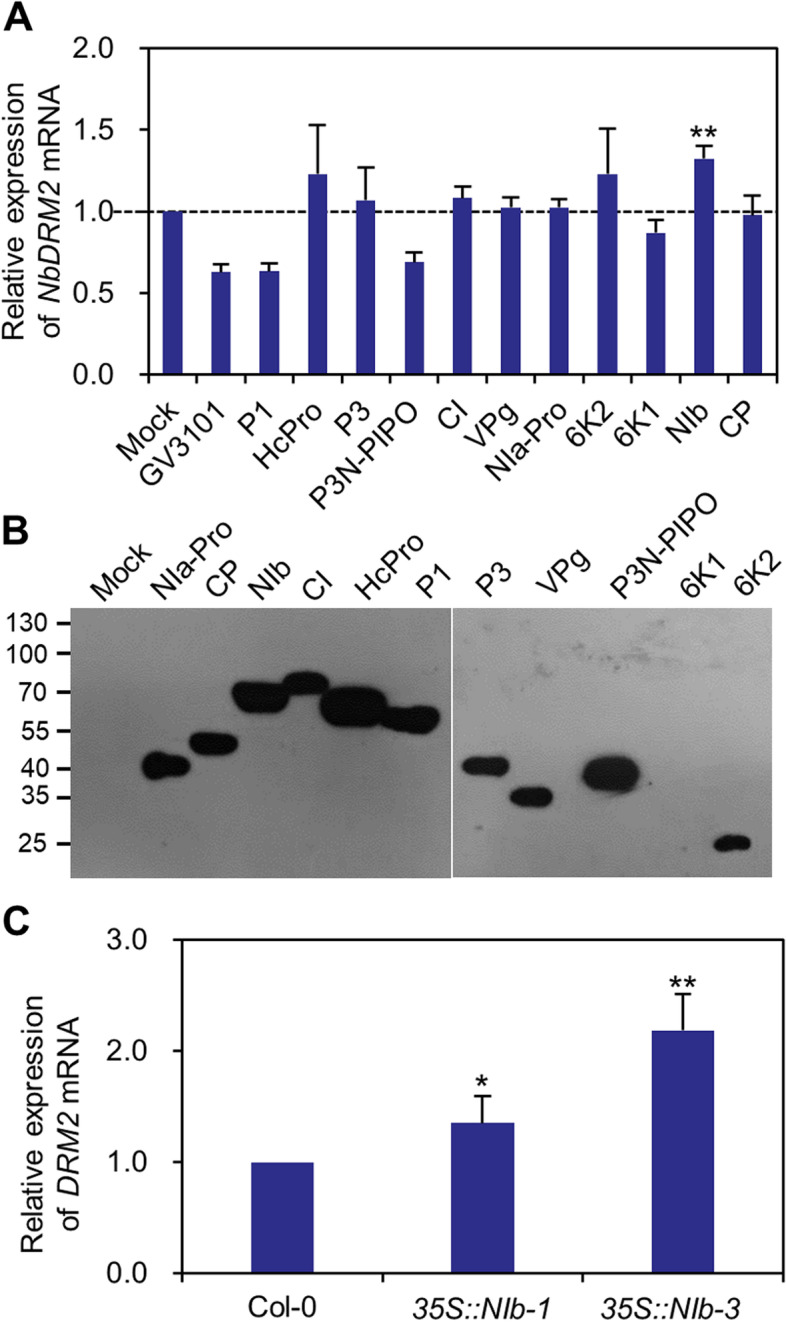
TuMV-encoded NIb induces the expression of *DRM2*. **A** RT-qPCR analysis the expression of *NbDRM2* in *N. benthamiana* epidermal leaves infiltrated with buffer (mock), empty *Agrobacterium* strain GV3101, or one of the eleven TuMV-encoded proteins. **B** Validation the expression of the eleven TuMV-encoded proteins by Western blot with anti-Myc antibodies. **C** RT-qPCR analysis the expression of *DRM2* in wild-type or two transgenic lines expressing FLAG-4 × Myc-tagged NIb under the *cauliflower mosaic virus* 35S promoter (*35S:NIb-1* and *35S:NIb-3*). *ACTIN II* was used as the internal control. Transcript level in wild-type plants was normalized to 1. * and ** indicate *p* < 0.01 and 0.001 of the student *t*-test, respectively. Data are mean ± s.d. (*n* = 7)

Through transient expression assay, we were able to identify the viral protein that was able to induce the expression of *DRM2* (Fig. 5). Using stable transgenic plants, we further confirmed that NIb has the capacity to induce *DRM2* expression. NIb is a multi-functional protein, which is not only the viral RNA-dependent RNA polymerase, but also interacts with several host factors for suppressing host defenses (Shen et al. 2020). Importantly, we recently found that NIb is posttranslationally modified by SMALL UBIQUITIN-LIKE MODIFIER 3 (SUMO3) in nuclei, and the modification is associated with its immunodepression activity (Cheng et al. 2017). However, how NIb regulates the expression of genes in the RdDM pathways and the correlationship between its immunodepression activity and ability to regulate the expression of genes in the RdDM pathways is unknown at the present. At present, we are trying to answer these key questions.

Given the important role of DNA methylation in plant immunity against RNA viruses, increase the host DNA methylation level to reduce the expression of defense-related genes surely benefits viral proliferation. From this point of view, it is possible that many RNA viruses have the ability to manipulate of host DNA methylation. The infection of *cucumber green mottle mosaic virus* (CGMMV) on *Citrullus lanatus* also induces methylation at early infection stage (48 hpi), while hypermethylation at late infection stage (25 dpi) (Sun et al. 2019). Moreover, the methylation-related genes including *CMT* and *DRM* of *C. lanatus* was significantly downregulated at early infection stage but upregulated at late infection stage, indicating that the dynamic methylation profile during CGMMV infection is related to the expression of those genes in the RdDM pathway (Sun et al. 2019). Since most, if not all, leaf areas are infected by CGMMV at the late infection stage (Sun et al. 2019), it is possible that host DNA methylation may be also modulated by CGMMV through regulating the expression of genes in the RdDM pathgy. Nevertheless, further investigations are needed to explore this possibility.

In conclusion, this study discovered that TuMV is able to modulate host DNA methylation by regulating the expression of *DRM2* to promote virus infection.

## Materials and methods

### Plant and virus materials

*A. thaliana* and *N. benthamiana* plants were grown in pots at 23 °C in a growth chamber under a 16/8-h photoperiod. The *drm2–2* mutant was obtained from the Nottingham Arabidopsis Stock Centre (NASC), NIb transgenic plants were described previously (Cheng et al. 2017). Virus was inoculated by *Agrobacterium*-mediated infiltration or mechanical inoculation as described earlier (Cheng et al. 2017).

### Nucleic acid extraction, sodium bisulfite treatment, and whole-genome bisulfite sequencing

Virus-infected leaf area was dissected under a portable UV light. The total DNA was extracted using the FastPure Plant DNA Iolation Mini Kit (Vazyme). About 100 ng DNA were treated by sodium bisulfite with the EZ DNA Methylation Gold Kit (Zymo Research) and subjected to library preparation with the TruSeq DNA methylation Kit (Illumina). In total, six libraries were prepared including three biological replicates for each condition. Libraries were sequenced by the Illumina sequencer NovaSeq 6000.

### Whole-genome sequence analyses

Reads from high-throughput sequencer were first filtered by FastQC v0.11.7 to remove low quality reads and then treated by Trimmomatic 0.39 to remove the adaptor. Reads were them mapped to Arabidopsis genome (TAIR10) and used for methylation calling using BisMark (v0.23.0). The cytosines with less than 3 coverage were discarded in following analysis to ensure reliability. DMRs were identified using R package methylKit (v1.18) in a 200- base pair (bp) sliding window and a 50-bp step-size, FDR ≤ 0.05 and at least 0.3-fold change in methylation level were required to define DMRs. Then DMR-associated genes (DMGs) were identified according to overlapping with gene positions (Araport11), upstream 2 kb of transcription start sites (TSSs) were included in gene positions as promoter. Gene ontology (GO) enrichment was performed using R package clusterProfiler (v4.0.5).

### Searching for possible RdDM sites targeted by TuMV-GFP

The sequence of TuMV-GFP was spliced into 24-nt fragments using a custom script to simulate vsiRNAs. BLAST alignment (blastn -task blastn-short -evalue 0.05) was carried out using these fragments against all transcripts from Arabidopsis genome annotation (Araport11). The resulted alignments with at most 4-nt mismatch were selected as possible RdDM targets.

### Transcriptome analysis

RNA-Seq data of *drm2* mutant, SA-treated, and mock-treated wild-type Arabidopsis were downloaded from the NCBI SRA database. DEGs were calculated using StringTie v2.1.1 and DESeq2 v1.36.0 with false discovery rate (FDR) cutoff of 0.05 (Pertea et al. 2015; Love et al. 2014).

### RNA extraction and RT-qPCR

Total RNA was isolated using the PastPure Universal Plant Total RNA Isolation Kit (Vazyme). Massager RNAs were reverse transcribed into complementary DNA (cDNA) by Oligo-dT_20_ with the HiScript III 1st Strand cDNA Synthesis Kit with gDNA wiper (Vazyme). RT-qPCR was performed in a 20 μL volume system, containing 4 μL of tenfold-diluted cDNA, 5 μM of each primer, and 1 × AceQ® Universal SYBR qPCR Master Mix (Vazyme). All primers used in the present study are listed in Supplementary Table 1. All experiments were repeated in triple.

### Western blotting

Leaf tissues were ground in liquid nitrogen and resuspended in 100 µL 1 × SDS-PAGE sample loading buffer [62.5 mM Tris–HCl pH6.8; 2% Sodium dodecyl sulfate (SDS; weight/volume); 10% glycerol (volume/volume); 5% β-mercaptoethanol (volume/volume); 0.5% bromophenol blue (weight/volume)] to extract total proteins. After boiled at 95 °C for 5 min, the crude protein extract was centrifuged at 12,000 g for 10 min at 4 °C, and separated by electrophoresis in 12% SDS–polyacrylamide gel. Proteins were then transferred to polyvinylidene fluoride (PVDF) membrane using a Trans-Blot® Turbo™ Transfer System (Bio-Rad). Recombinant protein was detected by polyclonal anti-Myc (Abcam) at 1:5000 as described (Cheng et al. 2017).

## Supplementary Information

**Additional file 1.**

**Additional file 2.**

**Additional file 3.**

**Additional file 4.**

**Additional file 5.**

**Additional file 6.**

**Additional file 7.**

**Additional file 8: Table S1.** Primers used in this study.

## Data Availability

Data are contained within the article or Supplementary Material.
