# Supplementary material for: Turnip mosaic virus manipulates DRM2 expression to regulate host CHH and CHG methylation for robust infection
Source: Stress Biol. 2022 Aug 4;2(1):29. doi: 10.1007/s44154-022-00052-3 (PMC10441925; doi:10.1007/s44154-022-00052-3)
Supplement: Supplementary file 8 — Additional file 8: Table S1. Primers used in this study. [file 44154_2022_52_MOESM8_ESM.docx]

**Supplementary Table 1 Primers used in this study.**

| Primer Name | Sequence (5′-3′) | Usage |
| --- | --- | --- |
| ACTIN2-rtF | CCGGTATTGTGCTGGATTCT | RT-qPCR |
| ACTIN2-rtR | TTCTCGATGGAAGAGCTGGT | RT-qPCR |
| TuMV-CP-rtF | CAGGTTTGACAGACGAGCAA | RT-qPCR |
| TuMV-CP-rtR | CCAGAGGTTCCAGCGTTTAC | RT-qPCR |
| EDS5-rtF | TTATTCGGTCCTTGGGCTGT | RT-qPCR |
| EDS5-rtR | CCGTTAATGATCGTTGCTGC | RT-qPCR |
| ICS1/SID2-rtF | CCTCCGTCGTTTTCCTCC | RT-qPCR |
| ICS1/SID2-rtR | CAGCGATCTTGCCATTAG | RT-qPCR |
| DRM2-rtF | CCCACCTGAGTTTGTGGACT | RT-qPCR |
| Drm2-rtR | CATTCTGGCCACCATCTCTT | RT-qPCR |
| MET1-rtF | CAAAAGGTCGTGCTTTCCGA | RT-qPCR |
| MET1-rtR | CCTCTGTCCCCATCCAATGT | RT-qPCR |
| CMT2-rtF | GACAGCGATGAACCGTCTTC | RT-qPCR |
| CMT2-rtR | CGCCATCTTTCTTGAGCCTC | RT-qPCR |
| ROS1-rtF | CATCGGCCAAAGGTTCGTAG | RT-qPCR |
| ROS1-rtR | CGGAGTAGCGTCTTGATCCT | RT-qPCR |
| CMT3-rtF | TTGACCTTTACTCCGGCTGT | RT-qPCR |
| CMT3-rtR | GCATAGCTTCTCCCACTCCT | RT-qPCR |
| ROS3-rtF | GCAAACACAGAGGGCGATAG | RT-qPCR |
| ROS3-rtR | CTAACCAGCTGAGTCCACGA | RT-qPCR |
| DML2-rtF | ATGTCGGACGCATAGCTGTA | RT-qPCR |
| DML2-rtR | CTCCGCCTTCATTGGACATG | RT-qPCR |
| DML3-rtF | CCTCGTTCGTTCCACCAAAG | RT-qPCR |
| DML3-rtR | CCCTCGCATTGCTGTTCTAC | RT-qPCR |
| PR1-RTF | CTCATACACTCTGGTGGG | RT-qPCR |
| PR1-RTR | TTGGCACATCCGAGTC | RT-qPCR |
| PR2-rtF | TCCGGTACATCAACGTTGGA | RT-qPCR |
| PR2-rtR | AAGGGAGATTGCTTGCTTGC | RT-qPCR |
| PR3-rtF | CCAATGCAACTGTCGTGGAA | RT-qPCR |
| PR3-rtR | CTATCACGGCATGGCAAGAC | RT-qPCR |
| PR4-rtF | CACGTGGGATGCTGATAAGC | RT-qPCR |
| PR4-rtR | CATCCAAATCCAAGCCTCCG | RT-qPCR |
| PR5-rtF | CTTGCCCCGACATGCTTAAG | RT-qPCR |
| PR5-rtR | CGTTTCGTCGTCATAAGCGT | RT-qPCR |
| GH3.12/PBS3-rtF | TTGCTCTGTGGCTTAGTCCA | RT-qPCR |
| GH3.12/PBS3-rtR | CTGCTAATTCAGGACGTGGC | RT-qPCR |
| NPR1-rtF | TGCAATTGCTCTCCAACAGCTTCG | RT-qPCR |
| NPR1-rtR | GCGGCTAAAGCGCTCTTGAAGAAA | RT-qPCR |
| *drm2–2* LP | AGATCGCTTCCAGAGTTAGCC | Genotyping |
| *drm2–2* RP | TTGTCGCAAAAAGCAAAAGAG | Genotyping |
| LBb1.3 | ATTTTGCCGATTTCGGAAC | Genotyping |
